# Supplementary material for: An optimised pipeline for parallel image-based quantification of gene expression and genotyping after in situ hybridisation
Source: Biol Open. 2018 Mar 13;7(4):bio031096. doi: 10.1242/bio.031096 (PMC5936060; doi:10.1242/bio.031096)
Supplement: Supplementary information [file biolopen-7-031096-s1.pdf]

SUPPLEMENTARY MATERIALS AND METHODS

DNA EXTRACTION AND IMAGE PROCESSING PROTOCOL

for

An optimized pipeline for parallel image-based quantification of gene expression and genotyping after *in situ* hybridization

Tomasz Dobrzycki, Monika Krecsmarik, Florian Bonkhofer, Roger Patient and Rui Monteiro

REAGENTS AND EQUIPMENT

- Stereomicroscope equipped with a digital camera and imaging software
- Fiji Software
- 0.2 ml PCR tubes
- glycerol
- forceps *or* P20 pipette and tips
- single- or multi-channel P200 pipette and tips
- BRAND® glass microscope slide with cavity for imaging embryos: L × W 76 mm × 26 mm, thickness 1.2-1.5 mm, with 1 concavity (BR475505, Sigma)
- HotSHOT alkaline lysis buffer (25 mM NaOH, 0.2 mM disodium EDTA, pH 12)
- HotSHOT neutralization buffer (Tris HCl 40 mM, pH 5)

PROCEDURES

Imaging

1. Prepare a glass slide with a drop of 100% glycerol.
2. Place the first ISH-probed embryo from 80% glycerol onto the slide and take a picture of the embryo with the appropriate orientation and magnification for the ISH staining. ** Save picture in TIFF format*
3. Transfer the embryo either with forceps or with a P20 pipette into a fresh 0.2 ml PCR tube with a minimum amount of glycerol.
4. Continue taking pictures of the remaining embryos using the same illumination, magnification and exposure settings.

DNA extraction

5. Add 40 µl of HotSHOT alkaline lysis buffer to the PCR tubes (from step **3**.) and make sure the embryos are in the solution. ** Using up to 75 µl does not affect the efficiency of the protocol.*
6. Incubate the tubes at 95°C for at least 5 minutes. ** Incubation longer than 65 min might reduce the success rate of the amplification of a PCR product.*
7. Cool the tubes to 4°C before adding an equal volume (40 µl) of HotSHOT neutralization buffer. ** Overnight incubation at 4°C after the neutralization improves PCR efficiency*. Spin down the samples before each use.
8. Use 5 µl of DNA lysate for PCR to genotype the embryos.

Image analysis

9. For image processing, open the image in Fiji and invert the image with “Edit▶Invert”. Then change the image type to 8-bit (“Image▶Type▶8-bit”). * *We have generated a Fiji macro that performs these actions simultaneously on all images in a directory.*

Fiji Macro

```
// Recursively converts images to 8-bit/invert starting in a user-specified
directory.
macro "conv"{
dir1 = getDirectory("Choose Source Directory ");
list = getFileList(dir1);
dirD = getDirectory("Choose Destination Directory ");
counter = 1;
setBatchMode(true);
for (i=0; i<list.length; i++)
    {
      path = dir1+list[i];
      if (endsWith(path, "tif")){
            print(path);
            run("TIFF Virtual Stack...", "open=path");
            run("8-bit");
            run("Invert");
            counter_converted=IJ.pad(counter,2);
            print(counter_converted);
            saveAs("Tiff", dirD+"conv"+counter_converted);
      close();
      i = i + 1;
      call("java.lang.System.gc");
      }
   counter = counter+1;
   i = i-1;
  }
}
```

10. Using the polygon selection tool, draw your region of interest (ROI) manually on the image around the region containing the signal.
11. Press “t” to open the ROI manager. Use the ROI manager’s Measure command to measure the intensity of the ROI. Copy the “mean” value from the Results window to Excel or equivalent calculation software.
12. Move the same ROI, ensuring the same size and shape as the original region, to a region of the zebrafish not containing any staining. Repeat step **11** to measure the background.
13. To obtain the mean pixel intensity of the ISH signal, subtract in Excel the mean intensity value of the background region from that of the stained region for each embryo.

Biology Open • Supplementary information

Statistical analysis

14. Assign each intensity value to a genotype (from step **8.**). * *If scoring phenotypes by eye (eg. 'high', 'medium' or 'low'), assign the phenotypes to genotypes and analyse with contingency chi-squared test.*
15. Plot all the values on one Q-Q plot to identify any deviations from normal distribution. * *Normal distribution can also be verified with the Kolmogorov-Smirnov test or the Shapiro-Wilk test. However, for large sample sizes there is a high risk of false positives in these tests.*
16. If there are strong deviations from normal distribution, transform all the values (using e.g. ln or sqrt functions) to make sure they are normally distributed before proceeding.
17. Analyse the differences between the values (transformed if necessary) assigned to each genotype (wt vs heterozygote vs mutant) with 2-tailed ANOVA with 95% confidence levels, accounting for the equality of variances with a Levene's test and Welch correction. For pairwise comparisons between each pair of genotypes, use Tukey's (equal variances) or Games-Howell (unequal variances) post-hoc test.
18. Plot the untransformed values (from step **14**.) as dot plots for the best representation of the results.

Biology Open • Supplementary information

Fig. S1

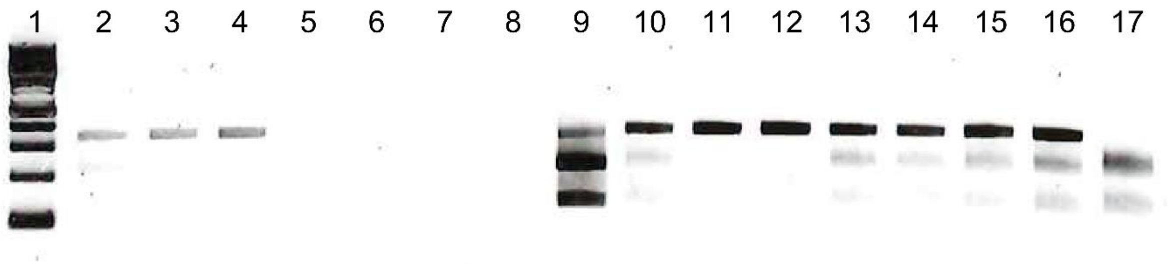

Figure S1. JumpStart™ REDTaq® ReadyMix™ performs more consistently on fixed ISH-probed genomic DNA samples than Phire™ Green HotStart II PCR Master Mix. 2% agarose gel showing digested PCR products amplified from seven randomly selected embryos at 33hpf from a *runx1*$^{+/W84X}$ heterozygote incross probed for *dnmt3bb.1*, plus an independent positive control (fin-clip from a wild-type 3dpf embryo, lanes 9 and 17). DNA fragments from the embryos were amplified either with Phire™ Green HotStart II PCR Master Mix (lanes 2-9) or with JumpStart™ REDTaq® ReadyMix™ (lanes 10-17). Lane 1: 100bp DNA ladder.

Biology Open • Supplementary information

Fig. S2

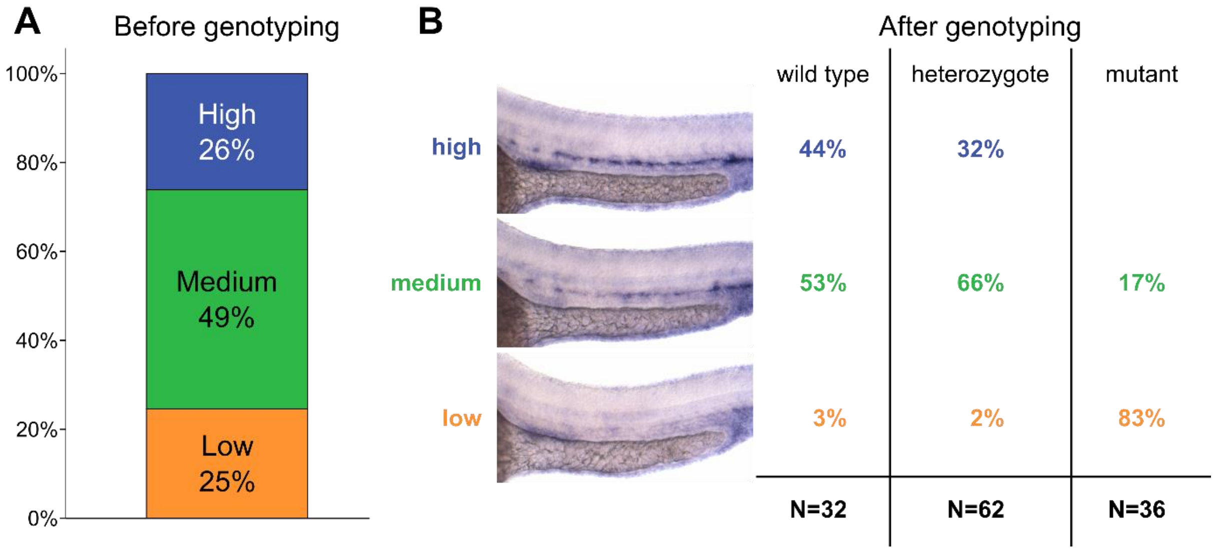

Figure S2. Phenotypic scoring of *dnmt3bb.1*-probed embryos does not fully correspond to their *runx1* genotype. A) The phenotypes of 130 33hpf *dnmt3bb.1*-probed embryos coming from the incross of *runx1* heterozygotes scored as ‘high’ (N=34), ‘medium’ (N=64) and ‘low’ (N=32) follow a Mendelian distribution. B) Distribution of the phenotypes according to the genotype. Images show a representative phenotype scored as ‘high’, ‘medium’ or ‘low’. Percentages show the occurrence of the phenotypes for the given genotype.

Biology Open • Supplementary information
